# Coronavirus disease-19 vaccine uptake, willingness for vaccination, and associated Factors among chronic follow patients attending in the two comprehensive specialized hospitals of Bahir Dar, Ethiopia

**DOI:** 10.1186/s12879-024-09882-0

**Published:** 2024-09-13

**Authors:** Dessie Tegegne, Mulu Kebede, Henok Biresaw, Astewle Andargie, Mengistu Ewunetu, Getenet Dessie

**Affiliations:** 1 Department of Medical Laboratory Sciences, College of Health Sciences, Debre Tabor University, Debre Tabor, Ethiopia; 2 Department of Medical Microbiology, College of Health Sciences, University of Gondar, Gondar, Ethiopia; 3 Department of Nursing, College of Health Sciences, Debre Tabor University, Debre Tabor, Ethiopia; 4 Department of Adult Health Nursing, College of Medicine and Health Science, Bahr Dar University, Bahr Dar, Ethiopia; 5 National Centre for Epidemiology and Population Health, College of Health and Medicine, Australian Capital Territory, Australian National University, Canberra, Australia

**Keywords:** COVID-19, Vaccine uptake, Willingness, Factors, Chronic follow-up

## Abstract

**Background:**

Even though the disease has spread throughout the world, with millions killed, global COVID-19 vaccination coverage remains low, particularly in developing countries. However, epidemiological data is lacking in the area. Hence, this study aimed to assess COVID-19 uptake, willingness for vaccination, and associated factors.

**Method:**

A hospital-based cross-sectional study was conducted from May 1 to June 30, 2022, among patients attending chronic follow-up clinics in the two comprehensive specialized hospitals in Bahir Dar. The total sample size was 423. Participants were selected by a systematic random sampling technique. Data was gathered using a pre-tested questionnaire and analyzed using SPSS version 23. A descriptive analysis was performed. A binary logistic regression analysis was done to assess the association between variables. Variables with a p-value < 0.05 in the multi-variable logistic regression with a 95% confidence interval were considered statistically significant.

**Results:**

The analysis included 400 out of 423 participants, representing a 95% response rate. The COVID-19 vaccination uptake was 46.8%, while the acceptance was 60.5%. About 56% and 68% of the respondents had good knowledge and a favorable attitude, respectively. Elderly people were 2.7 times more likely to be vaccinated. Similarly, urban residents were 3.94 times more vaccinated. The probability of being vaccinated among respondents with good knowledge and favorable attitudes was 70% and 79%, respectively. The willingness for vaccination increased among those individuals with favorable attitudes (AOR: 1.82). Urban people were less likely to accept vaccination (AOR: 0.46). Some participants misunderstood that vaccination may aggravate their disease condition.

**Conclusion:**

The overall COVID-19 vaccine uptake and acceptance for vaccination were low compared to what was estimated by the WHO. Age, residence, knowledge, and attitude were associated with COVID-19 vaccine uptake and acceptance of vaccination. Besides, there was a high level of rumor about the status of the vaccine and risk factors. Hence, special emphasis is warranted to deliver centrally trusted information. Moreover, further nationwide studies are warranted in the future.

## Introduction

The pandemic coronavirus was named coronavirus disease 2019 (COVID-19) by the WHO and began at the beginning of December 2019 near Wuhan City, Hubei Province, China [1]. It is an acute respiratory disease syndrome caused by the coronavirus-2 (SARS-CoV-2) that can spread rapidly with the increased emergence of new strains [2]. Despite the implementation of preventive measures, the burden of the pandemic is not significantly reduced [3].

The COVID-19 pandemic, caused by SARS-CoV-2, has resulted in over 774,771,942 confirmed cases and 7,035,337 deaths globally [4]. Africa is also heavenly affected by the pandemic, where more than 9,576,309 confirmed cases and 175,500 deaths were reported. COVID-19 also significantly affected Ethiopia, with 501,157 confirmed cases and 7,574 deaths [4, 5].

Although COVID-19 can infect all individuals, not all people are equally affected by the virus, develop the disease, and die [6]. Chronic follow-up patients, especially when they are unvaccinated, were more likely to progress to severe conditions and death [7–11]. A study conducted in the USA reported that more than 99% of deaths and 94.4% of hospital admissions related to COVID-19 occurred among unvaccinated high-risk individuals [9, 12].

In Ethiopia, one recent study indicated that the COVID-19 pandemic causes 72% of poor medication adherence, commonly linked to the impacts on their follow-up visits, availability of medications, and increased prices [13]. According to a recent study conducted in 2024 in Bahir Dar city, a total of 72 (17.4%) participants reported at least one side effect following the COVID-19 vaccination. The prevalence was higher in participants with chronic follow-up patients who had a history of regular medication use [14].

Community-level vaccine coverage of 65 to 80+% (average 70%) is required to protect the community from COVID-19 infection, which also depends on its coverage and public willingness for vaccination [15]. However, global COVID-19 vaccine coverage is inequitable and lagging, especially in developing countries [16]. In Australia, 81.5% had received at least one dose of the COVID-19 vaccine [17]. Similarly, in India, among cancer patients, 80% of COVID-19 vaccinations were reported [18]. In contrast, as of June 2022, only two countries in the African Region (the Seychelles and Mauritius) have achieved the 70% target [19].

On December 31, 2023, approximately 860 million doses of the COVID-19 vaccine had been delivered to countries in the African Region, and 646 million doses had been administered. Cumulatively, 38% of the African Region’s population had received ≥ 1 dose, 32% had completed a primary series, and 21% had received ≥ 1 booster dose. The total population coverage with ≥ 1 dose ranged by country from 0.3 to 89% [20]. Meanwhile, in Malawi (22%) [21], among DM patients in Sudan (31%) [22] had received at least one dose of the COVID-19 vaccine. According to the data reported on July 6, 2022, the vaccination coverage in Ethiopia reached 38.4% [23].

Availability and accessibility of a safe vaccine do not necessarily guarantee to mitigate the pandemic unless vaccine recipients are willing to utilize the vaccine [24, 25]. This might be due to public attitude and perception [26, 27]. According to different studies, the willingness for COVID-19 vaccination was (80–90%) in China, Brazil, and South Africa, and (50–60%) in Russia, Poland, and France [28, 29]. A study conducted in China reported that acceptance was 79.08% [30]. Similarly, acceptance was reported at 36.2% among DM patients in Saudi Arabia [31], and 70.1% in Uganda [32].

Although the impact of COVID-19 among chronic follow up patients is significant, research on COVID-19 vaccine uptake and willingness among chronic follow-up patients in Bahir Dar is lacking. Few studies were conducted in Ethiopia. However, they were conducted either among health care workers within the first month of the first vaccine announcement in the country where the quality and safety issues were the greatest issues or in different areas of the country [33–36]. Moreover, we tried to include the vaccine uptake and associated factors. Acceptance of new and available vaccines varied based on differences in sociodemographic, economic, political, and personal factors [33, 37, 38]. Addressing these barriers is crucial to attain maximum vaccine coverage and eradicate this pandemic [39]. Therefore, this study aimed to assess COVID-19 vaccine uptake, willingness for vaccination, and associated factors among patients attending chronic follow-up in the comprehensive specialized hospitals of Bahir Dar, Ethiopia, from May 1 to June 30, 2022.

## Conceptual-framework

**Fig. 1:**
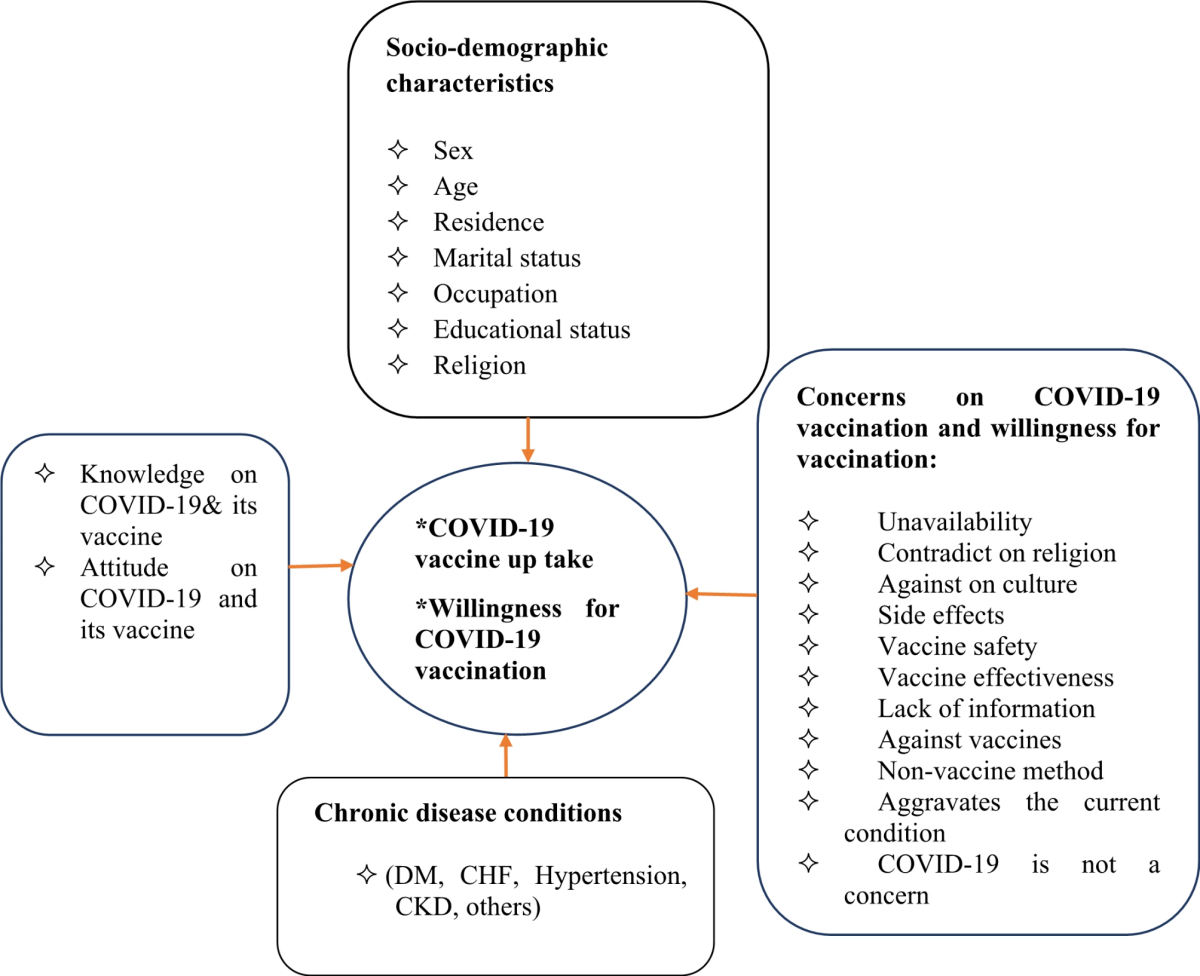
Conceptual framework of a study on COVID-19 vaccine uptake, willingness for vaccination, and associated factors among chronic follow-up patients in the two comprehensive specialized hospitals of Bahir Dar, Ethiopia; from May 1 to June 30, 2022

The conceptual framework was adapted after reviewing different literature [5, 8, 37, 42, 54]. This conceptual framework showed the effect of independent variables on the dependent variables (vaccine uptake and willingness) (Fig. 1).

## Methods and materials

### Study, design and period

A health facility-based cross-sectional study design was conducted from May 1 to June 30, 2022, in the two comprehensive specialized hospitals (Felege Hiwot Comprehensive Specialized Hospital (FHCSH) and Tibebe-Ghion Comprehensive Specialized Hospital (TGCSH)) found in Bahir Dar, Ethiopia. Bahir Dar is the capital city of the Amhara National Regional State, located about 570 km northwest of Addis Ababa, Ethiopia. The FHCSH is the largest comprehensive specialized hospital in the Amhara National Regional State, whereas the TGCSH is a teaching hospital affiliated with Bahir Dar University College of Medicine and Health Sciences. Each hospital serves about 700 patients per month, referred from various health facilities in Bahir Dar city administration and adjacent zones, who attend chronic follow-up clinics (including those with CHF, hypertension, DM, and other conditions).

### Source population and study population

The source population was patients with chronic diseases, including chronic liver disease, hypertension, diabetes mellitus, cardiovascular disease, chronic kidney disease, chronic obstructive pulmonary disease, asthma, and attending follow-up in the two comprehensive specialized hospitals. On the other hand, the study population consisted of patients who had chronic care appointments and follow-up during the data collection period, selected by the sampling technique and based on the inclusion criteria.

### Inclusion and exclusion criteria

All patients with chronic diseases who were volunteers to participate were included in the study. Patients with chronic diseases under the age of 18 and those patients who were unable to respond due to severe illness were excluded from the study.

### Operational definitions

Uptake: The percentage of people who received a COVID-19 vaccine, assessed by asking respondents if they had taken a vaccine (“Yes” or “No”) and reasons for not being vaccinated.

Willingness for Vaccination: The intention to receive available COVID-19 vaccines, assessed by asking respondents if they would take a vaccine if available (“Yes” or “No”). Reasons for refusal were assessed if the answer was “No.”

Chronic Disease Condition: Individuals with any medical illness, irrespective of the duration of follow-up.

Knowledge: Assessed using ten items about COVID-19 and its vaccine. Correct answers received one-point, incorrect answers received zero, and “I don’t know” responses were given negative one point. A score of 70% or above was considered good knowledge.

Attitude: Assessed using ten items. Agreement received one point, neutral zero points, and disagreement negative received one point. A score of 70% or higher was considered a positive attitude.

### Sample size determination

A single population proportion formula was used to calculate the sample size (n = Z α/22*P (1-P)/d2). Assumptions: Confidence level: 95%, Critical value (Z): 1.96, Precision (d): 0.05, Proportion for uptake (P): 0.05, Proportion for willingness (P): 0.59 [35].

Calculations:

Vaccine uptake: n = (1.96) ² * 0.5 (1-0.5) / (0.05) ² = 384.

Willingness: n = (1.96) ² * 0.59(1-0.59) / (0.05) ² = 372.

Final sample size: Using the larger sample size (384) and adding a 10% non-response rate, the total sample size was 423.

### Sampling technique

A systematic random sampling technique was used. The sampling frame included 1392 patients attending chronic follow-up, using the average monthly case flow of the previous year as a baseline. The sampling fraction (K) was calculated as 1392/423 = 3.29, rounded to 3. Patients were selected every 3rd interval from those actively involved participants during the data collection period. The first participant was chosen by lottery from the first three patients, and subsequent participants were selected systematically.

### Data collection tool and procedure

The primary data source was obtained through a direct face-to-face interview. The data were collected using a pretested questionnaire adapted from the previous relevant literature [5, 8, 37, 42, 54]. The questionnaire consisted of different sections that covered socio-demographic characteristics, questions related to vaccine uptake and willingness for vaccination, knowledge, attitude, and chronic disease conditions. Reasons related to previous un-vaccination and COVID-19 vaccine refusal were also included in the questionnaire. The data was collected by two nurses in each hospital under the supervision of one MSc holder.

### Data quality assurance

Training was given to data collectors and supervisors. Trained nurses who know the treatment centers were recruited. The questionnaire was translated to the local language, Amharic, and then back to the English version to assure its consistency during data recording and analysis. A pretest was also conducted on 5% of the sample size at Debre Tabor Comprehensive Specialized Hospital, and adjustments were made to the tool as necessary. The reliability of the questionnaire was also assessed. The principal investigator and supervisors were actively involved in supervising and monitoring the data collection. The completed questionnaire was checked daily for completeness and consistency. Selection bias might be expected during the data collection. To minimize this, we employed a prospective study design and provided training for data collectors recruited outside of the health facility of our interest.

### Data processing and analysis

During the data collection, the questionnaire was checked for its completeness. Unrecorded values or responses were manually cleaned up. Data was entered into the epi-data software version 4.6 and exported to SPSS version 23 for analysis. Descriptive analysis was done first to find the frequencies and percentages. Binary logistic regression analysis was done using the crude odds ratio (COR), and multi-variable logistic regression with an adjusted odds ratio (AOR) at the 95% CI was performed to assess the association between dependent and independent variables. The p-values < 0.25 and < 0.05 were taken as cut-off values to test the level of statistical significance of the association for the bi-variable and multivariable logistic regression analyses, respectively.

## Results

### Socio-demographic characteristics

A total of 400 patients (95% response rate) participated in this study. The mean age was 53.5 years (SD = 17.3). Most participants were from FHCSH (52.5%), female (56.5%), and unable to read and write (51.3%). The majority were married (74.3%), Orthodox Christians (81.3%), and of Amhara ethnicity (99.2%). The most common occupation was farming (41.5%), and the most common diagnoses were hypertension (26.2%), followed by diabetes mellitus (18.2%) (Table 1).

**Table 1:** Socio-demographic characteristics of the study participants among chronic follow-up patients in the two comprehensive specialized hospitals in Bahir Dar, Ethiopia; from May to June 2022

Socio-demographic variables		Frequency	%
Facility/institution/	FHCSH	210	52.5
	TGCSH	190	47.5
Age in years	18–25	32	8%
	26–33	38	9.5%
	34–41	43	10.75%
	42–49	45	11.25%
	50–57	54	13.5%
	58–65	65	16.25%
	66–73	66	16.5%
	> 73	57	14.25%
Sex	Male	174	43.5
	Female	226	56.5
Educational background	Unable to read and write	205	51.3
	Read and write only	47	11.8
	Primary education	56	14
	Secondary education	34	8.5
	College/University	58	14.5
Residence	Urban	196	49
	Rural	204	51
Marital status	Single	59	14.8
	Married	297	74.3
	Divorced	26	6.5
	Widowed	18	4.5
Religion	Orthodox Christian	325	81.3
	Muslim	71	17.8
	Protestant	4	1.0
Occupation	Farmer	166	41.5
	Merchant	60	15
	Housewife	83	20.8
	Government employee	56	14
	Others	34	8.7
Ethnicity	Amhara	397	99.2
	Tigray	3	0.8
Chronic disease condition	Hypertension	105	26.2
	DM	73	18.2
	CHF	59	14.8
	Kidney problem	59	14.8
	Both DM and hypertension	55	13.8
	Respiratory problem	49	12.2

### Participants knowledge about COVID-19

More than half (55.7%; 95% CI: 50.7–60.7%) had good knowledge of COVID-19 and its vaccine. The majority (84.5%) had information regarding the disease and its vaccine. Of those who had information, most (42.3%) considered television and radio as trusted sources of information. Most (67.5%) and 73.75% knew the transmission and the symptoms associated with COVID-19, respectively. The majority (75%) knew that going to crowded public places is a risk, while 66.25% knew that wearing a mask is necessary even after vaccination. Furthermore, 65.2% and 78% knew that chronic patients were among the priority groups for the vaccine and understood its availability for COVID-19, respectively (Table 2).

**Table 2:** Participants’ knowledge of COVID-19 and its vaccine among chronic follow-up patients in the two comprehensive specialized hospitals in Bahir Dar, Ethiopia, from May to June 2022

Variables/items used to assess knowledge		Number (%)
Have information on COVID-19 and its vaccine	No	62 (15.5)
	Yes	338 (84.5)
The source of information for those having information	Social media	39 (11.5)
	Television and radio	143 (42.3)
	Friends and families	50 (14.8)
	Health care workers	106 (31.4)
COVID-19 is transmitted via respiratory droplets in infected individual	I do not know	55 (13.75)
	No	75 (18.75)
	Yes	270 (67.5)
The main clinical symptoms of COVID-19 are fever, fatigue, cough, and breathing problem	I do not know	40 (10)
	No	65 (16.25)
	Yes	295 (73.75)
Chronic patients including you are among the high-priority groups for the vaccine	I do not know	13 (3.3)
	No	126 (31.5)
	Yes	261(65.2)
There is a vaccine for COVID-19	I do not know	26 (6.5)
	No	62 (15.5)
	Yes	312 (78)
The vaccine is provided for free in Ethiopia	I do not know	39 (9.7)
	No	69 (17.3)
	Yes	292 (73)
The provision of the vaccine is based on voluntary	I do not know	0 (0)
	No	0 (0)
	Yes	400 (100)
To prevent COVID-19 infection, individuals should avoid going to crowded places	I do not know	56 (14)
	No	44 (11)
	Yes	300 **(75)**
Vaccinated individuals should wear a mask	I do not know	40 (10)
	No	95(23.75)
	Yes	265 (66.25)
Overall knowledge status	Good	223 (55.7)
	Poor	177 (44.3)

### Participants attitudes toward COVID-19 and its vaccines

Most respondents (68%; 95% CI: 63.2–72.5%) had a favorable attitude towards COVID-19 and its vaccine. Specifically, 72.8% felt susceptible to COVID-19, 79% believed the vaccine was essential, and 76.5% agreed that it should be given to all. Additionally, 61.25% believed vaccines provide long-term immunity, 62.5% believed vaccines prevent complications, and the majority did not believe the vaccine contradicted their religion (73.7%) or culture (80.2%) (Table 3).

**Table 3:** Attitude toward the COVID-19 vaccine among chronic follow-up patients in the two comprehensive specialized hospitals in Bahir Dar, Ethiopia; from May to June 2022

Variables/items used to assess attitude		Disagree	Neutral	Agree
		Number (%)	Number (%)	Number (%)
You believe that you are susceptible to COVID-19		47 (11.7%)	62 (15.5)	291 (72.8)
COVID-19 can be prevented by the vaccine		14 (3.5)	60 (15)	326 (81.5)
Vaccines will help to provide long-term immunity		100 (25)	55 (13.75)	245 (61.25)
Vaccination will ease complications of the disease		56 (14)	94 (23.5)	250 (62.5)
COVID-19 vaccine is essential for you		16 (4)	68 (17)	316 (79)
COVID-19 vaccine should be given to all		19 (4.8)	75 (18.8)	306 (76.5)
COVID-19 vaccine does not affect my religion		41 (10.3)	64 (16)	295 (73.7)
COVID-19 vaccine does not affect my culture		14 (3.5)	65 (16.3)	321 (80.2)
COVID-19 vaccine saves you money and time		15 (3.8)	83 (20.7)	302 (75.5)
COVID-19 vaccine is the primary solution to prevent		17 (4.3)	62 (15.5)	321 (80.3)
Attitude status	Unfavorable attitude	128	32%	
	Favorable attitude	272	68%	

### Coronavirus disease-19 vaccine uptake and associated factors

Nearly half, 46.8% (95%CI: 41.8–51.8%), had taken at least one dose of the COVID-19 vaccine. Specifically, 30% had been fully vaccinated, while 16.8% had been partially vaccinated or had been informed to have another dose. According to the multi-variable logistic regression analysis, the odds of COVID-19 vaccine uptake were 2.7 (95% CI: 1.17–6.2) among the older groups (age > 64 years) and 3.94 (95% CI: 1.64–9.5) for those living in the urban area. Besides, those with good knowledge and a favorable attitude toward COVID-19 and its vaccine had 2.3 (95% CI: 1.18–4.5) and 3.8 (95% CI: 1.8–7.9) increased odds for vaccine uptake (Table 4).

**Table 4:** COVID-19 vaccine uptake and associated factors among chronic follow-up patients in the two comprehensive specialized hospitals in Bahir Dar, Ethiopia; from May to June 2022

Variables		COVID-19 vaccine uptake		COR (95% CI)	*p*-value	AOR (95% CI)	*p*-value
		Yes	No				
Age in years	18–64	102	175	1		1	
	> 64	85	38	**3.84 (2.44–6.04)**	**< 0.001**	**2.7 (1.17–6.2)**	*0.019
Sex	Male	90	88	1		1	
	Female	97	125	**0.25 (0.17–0.38)**	**< 0.001**	0.5 (0.24–1.06)	0.072
Educational background	Illiterate	92	113	1			
	Read and write only	21	26	0.99 (0.5–19)	0.9	0.57(0.18–1.81)	0.34
	Primary education	25	31	0.99 (0.55–1.9)	0.98	0.56(0.2–1.6)	0.27
	Secondary education	17	17	1.2 (0.6–2.5)	0.58	0.36(0.08–1.63)	0.18
	College and above	32	26	1.5 (0.8–1.7)	0.17	1.4 (0.24–1.3)	0.085
Residence	Rural	59	145	1		1	
	Urban	128	68	4.63 (3.03–7.06)	< 0.001	**3.94 (1.64–9.5)**	*0.002
Marital status	Single	31	28	1			
	Married	155	142	1.01 (0.6–1.8)	0.96	0.87(0.31–2.4)	0.79
	Divorced	18	8	0.5 (0.2–1.3;)	0.16	0.3(0.05–1.63)	0.16
	Widowed	9	9	1.1 (0.4–3.2)	0.85	0.45(0.08–2.6)	0.37
Religion	Orthodox Christian	148	177	1			
	Muslim	36	35	1.23 (0.7-2.0)	0.4		
	Protestant	3	1	3.6 (0.4–35)	0.3		
Occupation	Farmer	72	94	1		1	
	Merchant	32	28	1.5 (0.83–2.7)	1.89	0.7(0.21–2.27)	0.55
	Housewife	37	46	1.05 (0.6–1.8)	0.86	0.8(0.28–3.3)	0.7
	Gov. employee	38	18	2.76 (1.5–5.2)	0.002	0.8(0.36–11.9)	0.4
	Others	8	27	0.4 (0.17–0.90)	0.028	0.34(0.07–1.61)	0.17
Ethnicity	Amhara	185	212	1			
	Tigray	1	2	1.8(0.97–3.3;)	0.06	2.4 (0.86–6.7)	
Chronic disease condition	Hypertension	42	63	1		1	
	DM	32	41	1.17 (0.64–2.14)	0.6	1.03(0.38–2.8)	0.95
	CHF	32	27;2	1.78 (0.93–3.39)	0.08	2.23(0.83-6.0)	0.11
	Kidney problem	30	29	1.55 (0.82–2.95)	0.18	1.66(0.56–4.9)	0.36
	Both DM and hypertension	30	25	1.8 (0.93–3.48)	0.08	1.4(0.5-4.0)	0.52
	Respiratory problem	21	28	1.13 (0.57–2.24)	0.74	2.18(0.71–6.67)	0.17
Knowledge status	Poor	50	127	1		1	
	Good	137	86	**4.0 (2.65–6.2)**	**< 0.001**	**2.3 (1.18–4.5)**	*0.014
Attitude status	Unfavorable	35	93	1	1	**1**	
	Favorable	152	120	**3.4 (2.13–5.3)**	**< 0.001**	**3.8 (1.84–7.87)**	*<0.001

**Key**: * showed the presence of a significant association

**Fig. 2:**
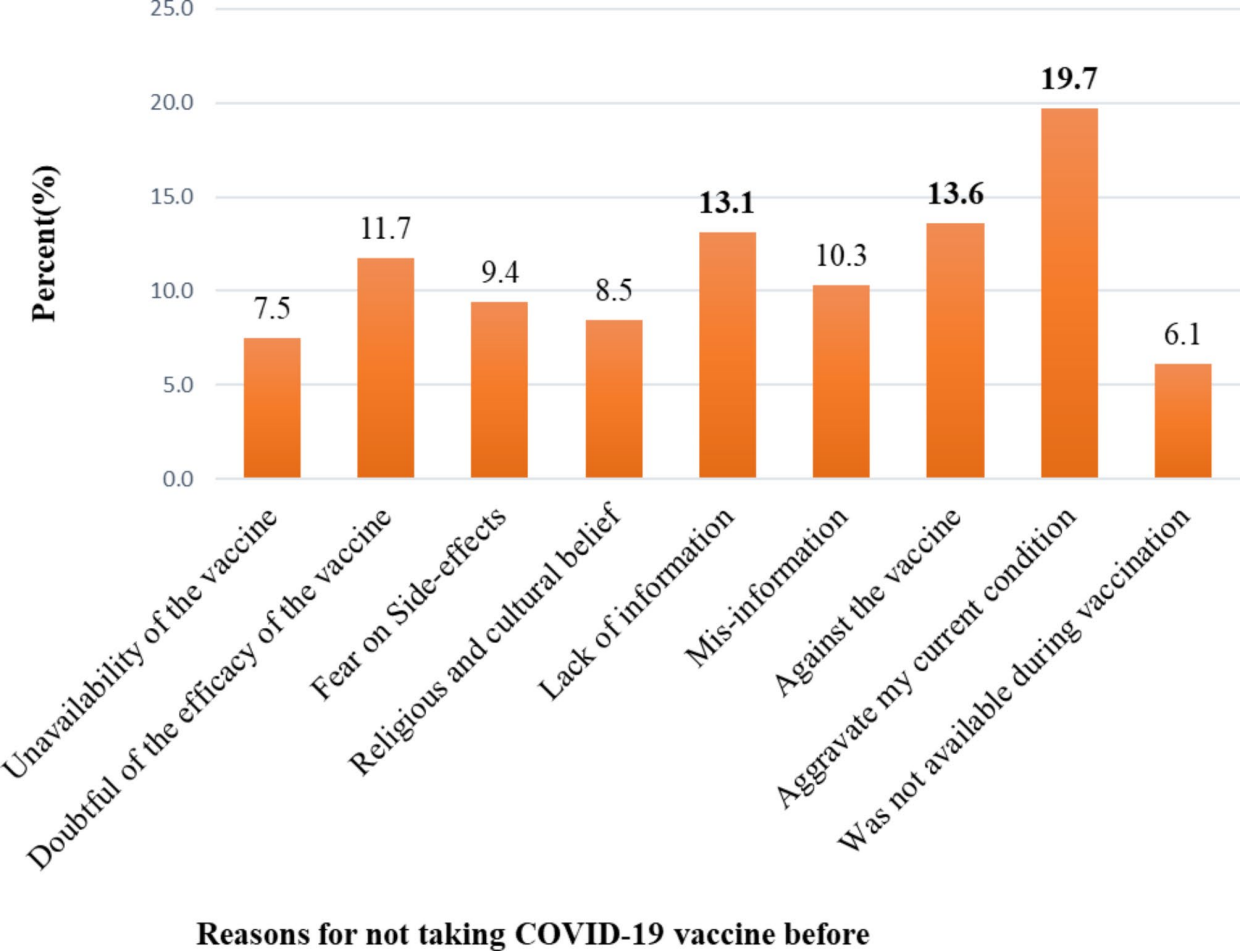
A bar graph showing respondents’ concerns or reasons for not taking the COVID-19 vaccine before among patients attending chronic follow-in the referral hospitals of Bahir Dar, Ethiopia; from May 1 to June 30, 2022

Respondents who had not taken the COVID-19 vaccine before (*n* = 213) were assessed for their concerns. Based on this, the majority (19.7%) worried that the vaccine might aggravate their current disease condition and treatment, which was followed by those who were against the COVID-19 vaccine, (13.6%) (Fig. 2).

### Willingness for the COVID-19 vaccination and associated factors

Of the total (*n* = 280) participants assessed for willingness for COVID-19 vaccination, the majority (60.5%; 95%CI: 54.5–66.3%) were willing to accept the vaccine if available. According to the multi-variable binary logistic regression analysis, participants who had a favorable attitude were more likely to accept the vaccine, with an AOR of 1.82 (95% CI: 1.03–3.21). The probability of accepting the vaccine among the urban population was lower (31.5%) with an AOR of 0.46 (95%CI: 0.22–0.92) (Table 5).

**Table 5:** The willingness for COVID-19 vaccination and associated factors among chronic follow-up patients in the two comprehensive specialized hospitals in Bahir Dar, Ethiopia; from May to June 2022

Variables		Willingness for vaccine (*n* = 280)		COR (95% CI)	*p*-value	AOR (95% CI)	*p*-value
		Yes	No				
Age in years	18–64	128	90	1		1	
	> 64	42	20	1.5(0.82–2.71)	0.19	1.5(0.71–3.1)	0.29
Sex	Male	67	30	1			
	Female	103	80	0.6 (0.36-0,99)	0.49		
Educational background	Unable to read and write	86	54	**1**		**1**	
	Read and write only	21	12	1.12(0.51–2.5)	0.78	0.95(0.37–1.83)	0.92
	Primary education	26	15	1.11 (0.54–2.28	0.78	1.45(0.6–3.47)	0.4
	Secondary education	18	10	1.15 (0.5–2.68)	0.74	1.24(0.39–3.96)	0.71
	College& above	19	19	0.64 (0.31–1.3)	0.22	0.34(0.08–1.5)	0.15
Residence	Rural	107	60	1		**1**	
	Urban	63	50	0.72 (0.44–0.8)	0.027	**0.46 (0.22–0.9)**	*0.043
Marital status	Single	27	18	1			
	Married	125	77	1.14(0.6–2.2)	0.69		
	Divorced	12	9	0.94(0.33–2.7)	0.91		
	Widowed	6	6	0.70(0.2–2.52)	0.59		
Religion	Orthodox	94	137	**1**			
	Muslim	16	30	1.28(0.66–2.7)	0.47		
	Protestant	1	2	1.4(0.12–15.2)	0.8		
Occupation	Farmer	75	38	1			
	Merchant	24	16	0.78(0.37–1.640	0.51		
	Housewife	37	27	0.71 (0.38–1.34)	0.29		
	Gov. employee	21	11	0.99 (0.44–2.77)	0.99		
	Others	13	18	0.38(0.17–0.85)	0.018	0.65(0.22–1.92)	0.43
Ethnicity	Amhara	170	109	1			
	Tigray	0	1	0.000	1		
Chronic disease condition	Hypertension	47	30	1		1	
	DM	24	25	0.63(0.31–1.3)	0.21	0.89(0.36–2.2)	0.8
	CHF	23	15	1.01(0.46–2.24)	0.98		
	Kidney problem	33	11	1.98(0.87–4.5)	0.103	1.18(0.46–3.05)	0.73
	DM& hypertension	24	12	1.32(0.58–3.02)	0.51		
	Respiratory	19	17	0.74(0.33–1.63)	0.45		
Knowledge status	Poor	89	55	1			
	Good	81	55	0.93(0.57–1.5)	0.75		
Attitude status	Unfavorable	53	51	**1**		**1**	
	Favorable	117	59	**1.95(1.19–3.19)**	**0.008**	**1.82(1.03–3.21)**	*0.039

**Key**: * showed the presence of a significant association

**Fig. 3:**
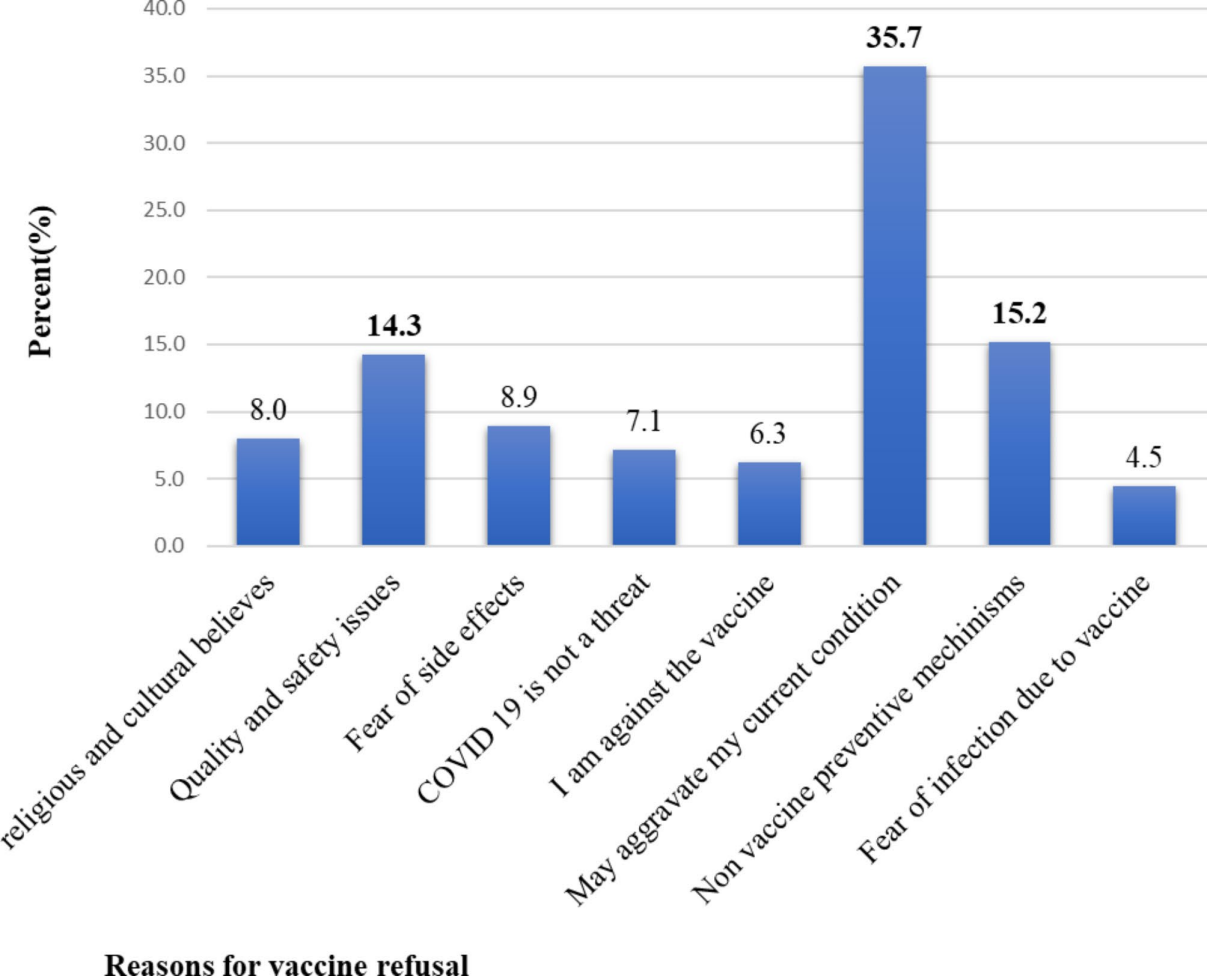
A bar graph showing respondent’s concerns or reasons for COVID-19 vaccine refusal among chronic follow-up patients in the two comprehensive specialized hospitals of Bahir Dar, Ethiopia; from May 1 to June 30, 2022

Out of 110 respondents who were assessed for their reason for vaccine refusal, most (35.7%) were due to their threat that the vaccine may aggravate their current disease condition or affect their treatment, which was followed by those who needed to apply non-vaccine preventable mechanisms (15.2%) (Fig. 3).

## Discussion

The present study found that 46.8% had taken at least one dose of the COVID-19 vaccine, 30% had been fully vaccinated, and 16.8% had been partially vaccinated. This implies that the vaccination was lower than what was expected by the WHO to achieve 70% vaccine coverage in the community [12, 15]. Hence, more than 50%, once exposed to COVID-19, might be vulnerable to increased COVID-19-associated morbidity, hospitalization, and mortality [9, 40].

Our finding was higher than a previous study conducted among cancer patients in India [18], a study done among NCD patients in Malawi by 2022 [21], and among DM patients in Sudan [22]. The discrepancy may be due to a difference in the period of data collection and the sociodemographic patterns of the study groups, as discussed below. For example, the study conducted in India only considered a specific group (only cancer patients aged ≥ 45 years), which might have affected the outcome. The data was also collected at the beginning of 2021, when the quality and safety of the vaccine were not assured and the awareness of the people was lower than reported elsewhere [41, 42].

Our finding also showed that the vaccine uptake in rural areas was lower (20.24%) than in the urban population (79.76%). This is in agreement with a study in Bangladesh [43]. This could be attributed to the vaccine’s inaccessibility in rural areas. It may also be due to rural people’s lack of awareness and religious or cultural beliefs about the virus and its vaccine. In support of this, a global survey conducted in 90 countries showed that Christianity was negatively related to the COVID-19 vaccination [44].

The odds of COVID-19 vaccine uptake were 2.7 among the older age groups (age > 64 years) as compared to the adult age groups (18–64 years). Participants living in the urban area were also more likely to be vaccinated than those living in the rural area, with an AOR of 3.94. Similarly, respondents with good knowledge and favorable attitudes towards COVID-19 and/or its vaccine were more likely to take it, with an AOR of 2.3 and 3.6, respectively. This implies that older people are more highly threatened by the disease than the adult groups, as the burden and severity have been considered high in the older age groups since the emergency of the pandemic [45, 46].

Due to increased accessibility and awareness in the first pandemic era, urban residents may have taken the COVID-19 vaccine more often. The same is true for the participant’s knowledge and attitude. Participants who did not take the COVID-19 vaccine were also assessed for their reasons or concerns for why they did not take the COVID-19 vaccine. The majority, 45 (19.7%) of the respondents, worried that the vaccine might aggravate their current disease condition or treatment, which was followed by those who were totally against the COVID-19 vaccine (13.6%). This indicated that, although the people had information, they either lacked awareness or were misinformed. Our finding is in agreement with a previous study showing the negative impact of misinformation and/or a rumor on the acceptance of the COVID-19 vaccine [47–50].

In the present study, 60.5% were willing to accept the vaccine if it was available. This implies that although people have information, either they do not understand or have been misinformed about the effectiveness of the vaccine or its safety [35]. Availability and accessibility of a safe COVID-19 vaccine do not necessarily guarantee to mitigate the COVID-19 pandemic unless vaccine recipients are willing to utilize the vaccine [24, 25]. The willingness to vaccinate can be affected by multiple beliefs and misconceptions among different population classes [35]. Our finding is in agreement with the previous study conducted in DCSH (59.4%) [35], and GCSH (63.8%) [51]. Our finding was also in agreement with the systematic review and meta-analysis studies conducted in Africa [52, 53] and around the world [54].

The willingness to accept the vaccine in our finding was higher than the study conducted among DM patients in Saudi Arabia, 36.2% [31], a study conducted among patients with NCD in rural areas of Malawi, 24% [21] and among patients with chronic disease in Guji Zone, Ethiopia, 39.5% [36]. The disagreement might be due to the difference in the period during which the studies were conducted. The perception regarding the quality and safety issues of the vaccine was misleading and in question during the first season of the pandemic, as reported in the previous studies [48, 49]. In contrast, our finding was lower than a study conducted among elderly and chronic patients in China, 79.08% [30]. The difference may be due to China’s high disease burden during the period when the pandemic first existed. It could also be due to China’s mandatory vaccinations. This finding was also lower than the study done in Uganda (70.1%) [32]. The source of this variation might be due to the study period where the burden of the pandemic was high in these areas, for example in China, initiating the people to accept the vaccine. It might also be due to the sociodemographic difference.

The probability of willingness for COVID-19 vaccination was higher among individuals with favorable attitudes. The implication is that the attitude or perception of the population matters more than the knowledge to accept new vaccines. This was in agreement with the previous study conducted in DCSH and Guji Zone, Ethiopia [35, 36].

In the present study, vaccine acceptance was higher among participants living in rural areas. In agreement with our finding, a study in China showed that urban participants had a higher COVID-19 vaccine hesitancy (9.39%) than their rural counterparts (4.26%) [55]. A study in Bangladesh also showed that the majority (84.3%) of the rural population responded to accepting the COVID-19 vaccine [56]. The increase in refusals for the COVID-19 vaccine in the urban population might be by chance and need further investigation. However, it could also be due to misinformation (“rumor”) delivered in the urban population by different social media about the vaccine’s status as reported elsewhere [48, 50, 55, 57, 58]. In support of this, a global survey reported that people’s perceptions toward COVID-19 vaccine acceptance have fluctuated with the information flow on various social media [59].

## Strength and limitation

This was the first study conducted to assess both COVID-19 vaccine uptake and willingness for vaccination among patients attending chronic follow-up who are one of the first WHO priority groups for COVID-19 vaccination. Besides, this study used probability sampling techniques, in which the results could be generalized to the population of chronic follow-up patients in the study settings. Despite this, during our data collection, HIV patients on ART follow-up and those cancer patients were attending a different clinic and were not included. This may underestimate the outcome and limit its generalizability. Causal relationships between the dependent and independent variables could not be established due to the use of a cross-sectional design. Furthermore, an interviewer bias might be expected. However, this had been managed through effective monitoring by the assigned supervisors in each of the hospitals. Hence, these limitations need to be considered when interpreting these findings.

## Conclusions

The overall COVID-19 vaccine uptake and willingness for vaccination were low compared with what was estimated by the WHO. Respondents’ age, residence, knowledge, and attitude towards the COVID-19 vaccine were all significantly associated with COVID-19 vaccine uptake. On the other hand, residence, and attitude towards COVID-19 and its vaccine were associated with willingness for vaccination. There was also a high level of misinformation or a rumor about the status of the vaccine and misunderstandings about the effect of the new vaccine on their underlining conditions. This could be an obstacle to the progress made in vaccine distribution and pandemic control.

Hence, special emphasis is warranted for individuals with chronic diseases through health education or information campaigns from trusted sources (health care workers, policymakers, and the media) on the safety of the current vaccine and its interaction with their underlining conditions. This is important to tackle the rumor, reduce its burden, and avoid the long-term impact of the pandemic on these high-risk groups. Moreover, future nationwide longitudinal and surveillance studies are warranted to monitor the public attitude, acceptance, and uptake of COVID-19 vaccination with the progress of vaccine improvement and the national vaccination program in terms of access, distribution, and coverage.

## Data Availability

The datasets supporting the conclusions of this article are included within the article. Materials relevant to this study can be obtained from the corresponding author (DT) for further inquiry.

## Abbreviations

AOR: Adjusted odd ratio
ART: Anti-Retroviral Therapy
COR: Crude odd ratio
CHF: Chronic heart failure
CI: Confidence interval
CKD: Chronic kidney disease
COVID-19: Coronavirus Disease 2019
DM: Diabetes mellitus
FHCSH: Felege Hiwot Comprehensive Specialized Hospital
GCSH: Gondar Comprehensive Specialized Hospital
HCW: Health care worker
ICU: Intensive Care Unit
MOH: Ministry of Health
NCD: Non-communicable disease
SPSS: Statistical Packages for socials Sciences
SARS: COV-2 Severe Acute Respiratory Syndrome COV2
TGCSH: Tibebe-Ghion Comprehensive Specialized Hospital
USA: United States of America
WHO: World Health Organization
